# Is the Bland-Altman plot method useful without inferences for accuracy, precision, and agreement?

**DOI:** 10.11606/s1518-8787.2024058005430

**Published:** 2024-02-09

**Authors:** Paulo Sergio Panse Silveira, Joaquim Edson Vieira, José de Oliveira Siqueira

**Affiliations:** I Universidade de São Paulo Faculdade de Medicina Departamento de Patologia São Paulo SP Brasil Universidade de São Paulo. Faculdade de Medicina. Departamento de Patologia. São Paulo, SP, Brasil; II Universidade de São Paulo Faculdade de Medicina Departamento de Cirurgia São Paulo SP Brasil Universidade de São Paulo. Faculdade de Medicina. Departamento de Cirurgia. São Paulo, SP, Brasil

**Keywords:** Confidence Intervals, Statistical Inference, Data Interpretation, Statistical, Regression Analysis

## Abstract

**OBJECTIVE:**

This study aims to propose a comprehensive alternative to the Bland-Altman plot method, addressing its limitations and providing a statistical framework for evaluating the equivalences of measurement techniques. This involves introducing an innovative three-step approach for assessing accuracy, precision, and agreement between techniques, which enhances objectivity in equivalence assessment. Additionally, the development of an R package that is easy to use enables researchers to efficiently analyze and interpret technique equivalences.

**METHODS:**

Inferential statistics support for equivalence between measurement techniques was proposed in three nested tests. These were based on structural regressions with the goal to assess the equivalence of structural means (accuracy), the equivalence of structural variances (precision), and concordance with the structural bisector line (agreement in measurements obtained from the same subject), using analytical methods and robust approach by bootstrapping. To promote better understanding, graphical outputs following Bland and Altman’s principles were also implemented.

**RESULTS:**

The performance of this method was shown and confronted by five data sets from previously published articles that used Bland and Altman’s method. One case demonstrated strict equivalence, three cases showed partial equivalence, and one showed poor equivalence. The developed R package containing open codes and data are available for free and with installation instructions at Harvard Dataverse at https://doi.org/10.7910/DVN/AGJPZH.

**CONCLUSION:**

Although easy to communicate, the widely cited and applied Bland and Altman plot method is often misinterpreted, since it lacks suitable inferential statistical support. Common alternatives, such as Pearson’s correlation or ordinal least-square linear regression, also fail to locate the weakness of each measurement technique. It may be possible to test whether two techniques have full equivalence by preserving graphical communication, in accordance with Bland and Altman’s principles, but also adding robust and suitable inferential statistics. Decomposing equivalence into three features (accuracy, precision, and agreement) helps to locate the sources of the problem when fixing a new technique.

## INTRODUCTION

Bland and Altman’s^1^ paper, which has become well-known and is widely used in various medical fields, introduced a graphical approach to compare two measurement techniques using peak flow meters. This method has been used to compare modern peak flow meters^2^, DNA sequencing methods^3^, athletes’ performances^4^, blood pressure measurements^5^, muscle tone quantifications^6^, and validations of self-reported height and weight^7^. It has been referenced in over 35,000 scientific publications.

In short, Bland-Altman plots assess the 95% limit of agreement (LoA) given by a band from the mean difference ±1.96 standard deviation of the measurements of two techniques. If the range between the lower and upper LoA is clinically unimportant, the techniques are assumed to be equivalent^8^. More recently, confidence intervals were added into the upper and lower LoA^11^ to provide a tolerance range. However, this tolerance only provides the band limits with a statistical test, not with an additional decision for technique equivalence. The Bland-Altman plot method is, therefore, subjective^16^. Clinical importance is attributable by the researcher as a threshold, in situations such as the acceptance of a null hypothesis only by visual inspection of the graph, without any inferential statistical support or measurement of the equivalence level.

Due to a lack of statistical support, the equivalence approach led to misunderstandings and anecdotal data interpretation, sometimes contradicting the original author’s recommendation. It is often misinterpreted that “two exams are equivalent when the majority of data are within the band limits,”^16,17^ which is always true, ranging from 75% to 100% independently of data distribution according to Chebychev’s inequality theorem^18,19^, or that “the points inside the band must be uniformly distributed,” which was never stated by the original authors. The Bland-Altman plot method is insufficient, as it only provides a visual decision.

Although widely used, the Bland-Altman plot method lacks a clear null hypothesis on method equivalence and, consequently, cannot guide statistical decision-making, and it relies on subjective judgment through visual inspection^16^. The available packages, in R language, are not sufficiently clear and do not provide a comprehensive solution to determine when two measurement techniques can be considered equivalent.

Our study applied a three-step statistical decision, allowing the researcher to determine if there are enough elements to reject the equivalence of two techniques. The solution includes three nested tests with *p-*values and robust statistical decisions by bootstrapping. This method was implemented into a freely distributable R package and the whole analysis, including statistics and graphical outputs, only requires one command line to be executed by the researchers.

## METHODS

This investigation proposes the addition of statistical criteria to Bland and Altman’s plot method^1^. Since it is a purely theoretical approach, it was not submitted to an ethics committee.

The R package containing open codes and sample data is available for free and with installation instructions on the Harvard Dataverse^20^.

### Rationale

We propose three steps to claim strict equivalence between measurement techniques: checking (1) the equivalence of structural means (equality of accuracy), (2) the structural variances (equality of precision), and (3) the agreement with the structural bisector line (equal measurements obtained from the same subject). Full equivalence can be assumed when none of the tests reject equivalence. This study considered a 5% significance level.

At first, this statistical approach may seem somewhat convoluted because it occurs when researchers have only observed the data, and decisions depend on structural, non-observable values. The obscure term ’structural’ refers, in this context, to true values, estimated from a statistical approach necessary to purge observed measures from measurement errors^21,22^.

Regressions applied to all three tests are not crude, but rather statistical artifices that connect structural values with functional procedures, providing conclusions on accuracy, precision, and agreement. This approach combines scattered statistical theoretical results from 1879 to 2015^11,23^. The tests are conceptually nested and propose inference based on solid mathematical foundation. The final test, which is also the most important one, assesses agreement with the bisector, demonstrating the reliability of the values obtained from the two measurement techniques applied to each individual. This test depends on the Deming regression^11,16^, which had its basic theorem developed over a century ago^28^. However, it would not make sense to authenticate such agreement if the two methods did not measure with equal precision—test 2, based on the theorem demonstrated by Shukla in 1973^24^—and nor with the same accuracy, introducing a bias—test 1, based on Hedberg and Ayers in 2015^23^.

Bootstrapping^30^ was also used to compute confidence intervals in addition to analytical tests. It was shown in graphics to support the researcher’s interpretation and to make it easier to communicate results. In our application, bootstrapping was represented by shadowed areas containing 95% of all resampled regressions, which is assumed to be the area containing the true populational regression.

The main concepts, balancing the connection between structural null hypotheses and their functional correspondences, are outlined in the following topics.

### Observed and true variable values

Measurements provided from a reference technique *A* and candidate under assessment technique *B* (each technique was applied once to each subject), according to the physics error theory, resulted in:


$$\begin{array}{rl} & B:y=Y+\delta \\ & A:x=X+\epsilon \end{array}$$ (1)

in which

y and x …are independent pairs of observed measurements,

Y and X …are the true correspondent measurements,

δ and ϵ …are independent measurement errors with a null average.

These error terms appear because all measurement techniques have a certain degree of imprecision. Assuming that Y and δ, and X and ϵ are also statistically independent and that these errors have no preferential direction (null averages, E[δ] = 0 and E[ϵ] = 0), the mean of all observed values is equal to the mean of the true values ( $\bar{y}=\bar{Y}$ and $\bar{X}=\bar{X}$ ), which is demonstrated by their respective expected (E) values:


$$\begin{array}{rl} & E[y]=E[Y+\delta ]=E[Y]+E[\delta ]=E[Y]\\ & E[x]=E[X+\epsilon ]=E[X]+E[\epsilon ]=E[X] \end{array}$$ (2)

Consequently, the observed mean difference between techniques is also equal to the structural bias ( $\bar{y}-\bar{x}=\bar{Y}-\bar{X}$ ). These equalities allow the functional computation and structural hypotheses to correspond, reducing all three nested tests to two ordinary least square linear regressions and one Deming regression. The relationship between structural and functional tests will be described in the following topics.

### Test 1: accuracy

In the analysis of covariance (ANCOVA), Hedberg and Ayers applied a covariate with measurement error to test mean structural equality for these repeated measure designs^23^. This simple linear regression applies the differences between measurements obtained from the same subjects, y_i_ − x_i_, and the centered value of the reference measurement, x_i_ − x–.

The null hypotheses:


$$H_{0.1}:E[X]=E[Y]H_{0,1}:\alpha =0y_{i}-x_{i}=\alpha +\beta \left(x_{i}-\bar{x}\right)+v_{i}$$ (3)

in which ν_i_ is the error term.

By centering values on the x axis, by subtracting x– from each original value, x_i_, the intercept of a regression line, α, becomes more meaningful as it corresponds to the mean of y−x , while the slope, β, is not affected. This artifice allows us to assess the equivalence of measurement means from different techniques, with the intercept representing the mean difference. Analytically, the null hypothesis of no mean difference is not rejected when zero is in the 95% confidence interval of the intercept.

Graphically, the regression intercept is the mean of y−x and located where the line crosses the y axis. The null hypothesis is (0, 0), meaning no difference between techniques. If bootstrapping shows (0, 0) outside the 95% confidence interval, the null hypothesis is rejected.

### Test 2: precision

The verification of equal variability of measurement errors in two techniques is based on Shukla^24^ and was also independently adopted by Oldham^26^ without widespread application. The null hypotheses are:


$$H_{0,2}:\lambda =V[\delta ]/V[\epsilon ]=1H_{0.2}:\rho (x-y,x+y)=0y_{i}-x_{i}=\alpha +\beta \left(x_{i}+y_{i}\right)+\theta_{i}$$ (4)

in which θ_i_ is the error term.

The structural null hypothesis computes lambda as the ratio between the variability of measurement errors. If the variability of errors is similar (λ = 1), the precisions of both techniques are similar.

It was demonstrated that a regression of y−x against x+y can detect unequal precisions, as the slope of the regression will not be null when the true value of λ ≠ 1^24,31^. Analytically and graphically, the null hypothesis of equal precisions is rejected if a horizontal line cannot be fitted into the 95% confidence band defined by the functional regression. Note that when each technique is applied to each subject more than once, it requires correction for computing λ —which was implemented according to the NCSS Manual^32^. The axes proposed by Shukla are the same ones used in Bland and Altman’s original concept^1^, which shows that the original method only compares the precision between measurement errors and is not a full equivalence test.

### Test 3: bisector line agreement

This test applies the Deming regression to verify if two measurement techniques measure the same values in the same subjects^11,25,27^. While the ordinary least square regression considers the independent variable *x* as free of measurement error, the Deming regression reasonably takes errors in both measurement techniques into account. Linnet^27^ studied several regression methods, showing that the Deming regression is robust and performs better than the ordinary least square regression.

When true values measured by two techniques coincide, ordered pairs of these measures follow the true bisector line. Therefore, the null hypotheses are:


$$H_{0,3}:Y=XY-X=\alpha +(\beta -1)X\alpha =0\beta =1H_{0,3}:E[y]=xy_{i}-x_{i}=\alpha +(\beta -1)x_{i}+\delta_{i}-\beta \epsilon_{i}$$ (5)

in which δ_i_ and ϵ_i_ are error terms.

Contrary to the ordinary least square regression statistical, which considers β = 0, the Deming regression verifies if the slope of the regression line is equal to 1 (β = 1), which represents the bisector line agreement. In addition, β simultaneously appears as the regression slope multiplying x and as part of the regression overall error term ( δ−βϵ ). Transitively, it implies that x becomes correlated with the combined error, preventing the computation of an ordinary least square regression^21,22,33^_._

The Deming regression also depends on λ, estimated in the previous step, to compute the true X and Y values before the computation of the regression estimates. When the value of lambda is not assumed to be 1, it affects the band width. Analytically, the null hypothesis is rejected if α ≠ 0 and β ≠ 1. Since these two parameters are estimated together, the Bonferroni correction is applied to control the probability of a type I error and preserve test power (effective significance level is 2.5%). Graphically, two alternative statistical approaches were implemented for the bisector line agreement: the assessments of the 95% prediction ellipse and of the 95% confidence band of regression, both done by bootstrapping. In the first one, the null hypothesis is to be rejected if (β, α) is not inside the ellipse, in the second one, if the bisector line cannot fit inside the band. These methods test intercept and slope together and provide stronger statistical power than an independent assessment of these two factors.

### Translations

**Figure 1:**
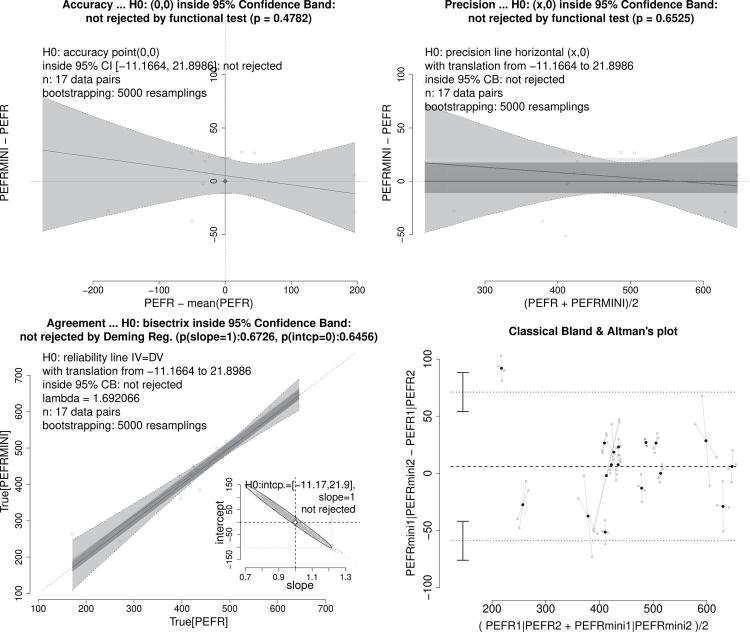
Graphical representation from accuracy, precision, and bisector concordance tests showing that peak flow measurements from Wright and Mini PEFR are strictly equivalent. See text, case 1. A traditional Bland-Altman plot is depicted for comparison with the precision test.

**Figure 2:**
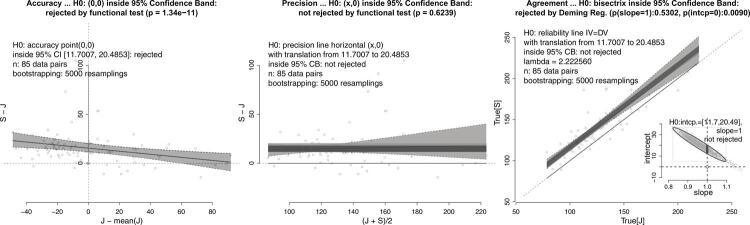
Comparison of systolic blood pressure measured by a human observer J and an automatic machine S showing a structural bias (overestimation by S) at accuracy test, and concordance at the precision test and bisector test. See text, case 2(a).

The three tests were conducted using both analytical (based on *p* value) and graphical (based on bootstrapping) approaches. Due to differences in accuracy, there are cases in which the analytical approach indicates no rejection of the null hypothesis while the graphical approach shows lines outside the confidence bands during precision and bisector line agreement tests. This discordance can be attributed to bias in a particular technique, as shown in the examples of Figures 1 and 2 (which are, respectively, cases without and with bias). Therefore, a combination of analytical and graphical approaches is necessary for better interpretation of precision and agreement^36^, especially in the presence of biased means.

The bias in accuracy can be corrected by translating lines according to the amount of bias computed. This correction enables the analytical approach to align with the graphical one, positioning lines inside the confidence band obtained by bootstrapping. For a precision test, the null hypothesis is not rejected when a horizontal line shifted by the bias can fit into the 95% confidence band (as shown in the examples of Figure1 [top-right panel] and Figure 2 [central panel]). Similarly, in the bisector agreement test, non-rejection of the null hypothesis occurs when the lines that are parallel to the bisector line, translated by the bias range, can fit into the 95% bootstrapping confidence regression band (as shown in the examples of Figure1 [bottom-left panel] and Figure 2 [right panel]).

## RESULTS

We revisited five data sets: one from the original Bland and Altman data^1^ (case 1), three from Bland and Altman^37^ (case 2), and another one from data provided by Videira and Vieira^38^ (case 3).

### Case 1

Bland and Altman^1^ proposed a graphical plot method which aimed to assess the equivalence of two peak expiratory flow rate (PEFR) measurement techniques: the Wright Peak Flow and Mini Wright Peak Flow meters. Our study, which involved 17 subjects, considered both of these instruments to be strictly equivalent. Figure 1 displays several statistical tests, including accuracy, precision, and bisector concordance, with p-values of 0.4782, 0.6525, 0.6726 and 0.6456, respectively. The structural regression bands were obtained by bootstrapping. Results show the null hypothesis inside the 95% confidence interval for accuracy, within the 95% confidence band defined by the structural regression for precision, and also inside the 95% confidence band defined by Deming regression for bisector concordance (λ = 1.692). Additionally, the bottom-left panel shows the 95% prediction ellipse, an alternative way to test slope and intercept together. A traditional Bland-Altman plot was also included for comparison; note that the axes are the same as those used in the precision test.

### Case 2

Bland & Altman^37^ provided three other examples of ways to apply their graphical method.

**Figure 3:**
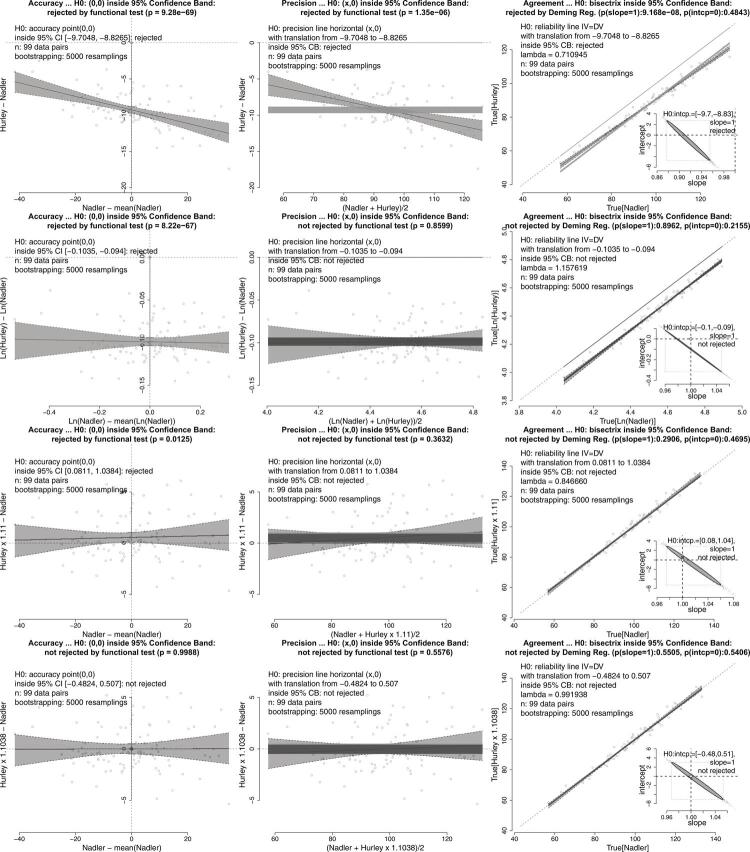
Comparison of the percentage of plasma volume in blood provided by two different equations (Nadler and Hurley methods) using raw data (upper panels), logarithm transformation (second row), Hurley measurements x 1.11 (third row), and Hurley measurements x 1.1038. See text, case 2(b).

**Figure 4:**
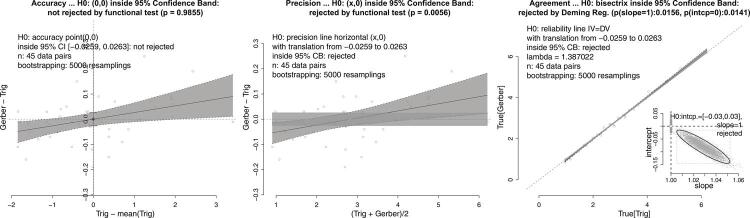
Comparison of the content of fat in human milk measured by glycerol released by enzymic hydrolysis of triglycerides (Trig) and by the standard Gerber method. These two methods measured equal average (left panel), but are not strictly equivalents in precision (central panel) or by bisector concordance (right panel). See text, case 2(c).

(1) In a comparison between systolic blood pressure measurements taken by an observer and an automatic machine, a systematic bias towards the machine was detected (n = 85). While the authors concluded that equivalence could not be assumed in this case, due to a large interval range, our analysis showed that the observer and machine may be interchangeable after deducting the bias. The structural bias is represented by the 95% confidence interval above the diamond, but the measurements passed precision and bisector line agreement tests. The intercept is inside the 95% prediction ellipse, and the non-null intercept cannot be corrected by traditional analytical approaches (Figure 2).

(2) The second example compares how two techniques, Nadler and Hurley, estimate the percentage of plasma volume in blood (n = 99). The original authors found increasing bias towards Nadler’s technique, since it had greater average values. Two strategies were then proposed to verify equivalence: logarithm transformation and scaling Hurley multiplied by 1.11. Figure 3 shows our approach, which confirms no equivalence between methods in any of the three tests (Figure 3, upper row). Logarithm transformation does not solve structural bias but leads to equivalences in precision and agreement lines (Figure 3, second row). The multiplication of Hurley values by 1.11 is a more successful strategy, with marginal failure for accuracy (Figure 3, third row). Using our approach, we found strict equivalence after multiplying Hurley values by approximately 1.1038, resulting in improved precision and agreement line tests (Figure 3, lower row).

(3) Bland and Altman^1^ compared fat content in human milk (n = 45) using enzymic hydrolysis of triglycerides and then the standard Gerber technique. They found that one technique overestimated smaller values and underestimated greater ones, thus requiring traditional lines to be adjusted into a slanting band formed by two straight lines, to accommodate these differences. Our proposal (Figure 4) naturally produced a slanted band, making adjustments unnecessary. Our results contradict the authors’ conclusion that the two techniques are equivalent in precision and agreement.

### Case 3

**Figure 5:**
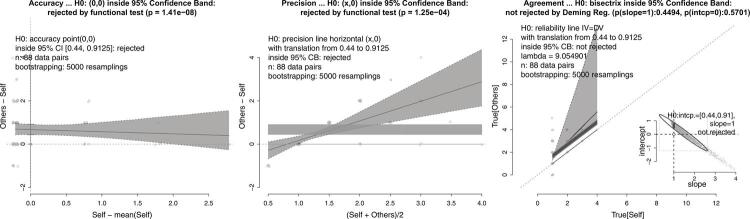
Graphical representation of anesthesiologists’ self-perception and peers’ perception about their skills in deciding on the use of neuromuscular blocking drugs showing no equivalence in accuracy and precision. See text, case 3.

Using questionnaires, Videira and Vieira^38^ compared anesthesiologists’ self-perception with their peers’ perceptions regarding their skills in deciding on the use of neuromuscular blocking drugs (n = 88). They found that self-perception and peer perception did not match; the subjects overestimated their abilities compared to their colleagues. Our approach (Figure 5) identified this bias as the “above-average effect” (tendency to consider oneself as better qualified) and also shows that the two perceptions are not equivalent.

## DISCUSSION

Bland and Altman’s analysis emphasizes clinical significance, and their plots largely ignore statistical inference, relying on visual inspection to draw what Watson and Petrie regard as subjective conclusions^16^. Our contribution adds an objective statistical inference to this method and locates causes of non-equivalence by isolating accuracy, precision, and bisector agreement, but it still follows the original Bland and Altman idea, preserving graphical outputs that facilitate communication^10^.

Altman and Bland argued that “the use of correlation is misleading” and insufficient for comparing clinical measurements^39^. They also emphasized that “comparability of techniques of measurement is an estimation problem: statistical significance is irrelevant”^40^. We respectfully disagree from the latter statement because it is necessary to compare related measurement techniques with proper confidence intervals. In fact, we looked for statistical treatment comparing any two related measurement techniques and for a proper method to compute confidence intervals, to replace non-informative Chebychev’s intervals with or without additional LoA flexibilization or adaptations, which create the slanted limits of agreement that these authors erroneously proposed^41^.

In this study, we analyzed five published data sets using the Bland and Altman plot method. The three-step tests we proposed implemented statistical support and were able to locate the sources of non-equivalence between techniques. In the case of peak flow expirometers^1^, for instance, we found that there was strict agreement in accuracy, precision, and agreement line. The other three data sets^37^ are examples of solvable equivalence between methods; our three-step tests, however, provide solutions more effectively. Finally, for the data set of Videira and Vieira’s^38^study, our approach showed the conceptual importance of nested tests, demonstrating that correcting the bias alone to assess the mean difference would result in a meaningless decision from the bisector line (third test) due to discrepancies in precision (second test). Without taking into account the nesting nature of our approach, one could have accepted equivalence despite the differences in precision.

There are other packages that address the Bland-Altman plot method in R: blandr, MethComp, MethodCompare, and mcr. The blandr^42^ package provides various ways to display the traditional plot, including limits of agreement (LoA) confidence intervals. Just like in the original method, the decision in blandr is solely based on visual inspection, and the Deming regression, which we consider to be the fundamental part for assessing complete equivalence between two measurement methods, is not applied. Interestingly, the example in the blandr.method.comparison function, from the blandr package, states that “Paired t-tests evaluate significant differences between the means of two sets of data. They do not test agreement, as the results of a t-test can be hidden by the distribution of differences,” “Correlation coefficients only tell us the linear relationship between 2 variables and nothing about agreement,” and “Linear regression models are conceptually similar to correlation coefficients, and again tell us nothing about agreement.” However, despite these correct statements about the limitations of these statistics, all three insufficient statistics were still computed in the example.

MethodCompare^43^ is a package with a small set of functions and aims to compare bias with precision. The author of this package, Patrick Taffé, was cited in this article^9,14^, and he aims to improve the confidence intervals of limits of agreement. Respectfully, we believe that studies focused on LoA^11^ do not address the fundamental issue of equivalence between methods. They are improvements of a secondary aspect of the Bland-Altman plot method, which already faces the problem of dealing with an uninformative interval, as discussed below.

MethComp^44^ implements several maneuvers accumulated in the literature in an attempt to improve the Bland-Altman plot method. This package uses the Passing and Bablok regression (PBreg), a non-parametric regression that has the same problem as the OLS regression: it does not consider the measurement error of one of the variables into account and, therefore, is also not the appropriate solution. This package has several functions with large numbers of parameters. Although it is evident that the author invested a lot of time and care in developing the package, there are no obvious tests to verify the bias or precision of its methods, and these evaluations are necessary for the Deming regression. Although this package includes the Deming regression, it does not display it with confidence bands and focuses on comparing it with the OLS regression, which (as the original authors of the Bland-Altman plot method themselves state^39^) should not be chosen. The parameters of the package request the value of lambda, with a default of 1, but do not provide resources to estimate it. Additionally, intercept and slope are not estimated together, which may incorrectly lead to the non-rejection of equivalence. This issue is illustrated in the second row of Figure 3. There is a small dotted rectangle drawn around the elliptical region of the agreement test. If the slope and intercept are not considered together, any point within this rectangle (i.e., any slope within its left and right limits, and any intercept within its bottom and top limits) would result in a statistical decision of non-rejection of the null hypothesis. However, the non-rejection decision should only be made when the point (1,0) is within the elliptical region. In the example given, non-rejection was only defined due to translation. It is important to note that without considering translation, MethComp would not be able to detect that the Deming regression line did coincide with the bisector.

We identified the package mcr^45^ as the closest one to ours. However, its approach also has limitations. This package includes a simple Bland-Altman plot without limits of agreement (LoA) using the plotDifference function. It also includes the Deming regression with the mcreg function. However, similar to MethComp package, it uses a default lambda value of 1 without providing guidance on its estimation, which is an issue. The approach to bias in the mcr package is somewhat incomplete and misguided. It uses the observed bias (y - x) as the dependent variable and the observed reference measurement values as the independent variable, without centering it by its mean value (x − x-). Then, as shown in the examples of the plotBias function, the mcr package lacks a clear statistical test for decision-making and uses several variations of the Deming regression, which should be performed with true values rather than observed values. The documentation of this function and of its regression variants is obscure. The author was apparently unaware of the theoretical foundation of Hedberg and Ayers^23^, which could have been used to develop a statistical test for accuracy. Furthermore, the package has no functions to verify the equivalence of precisions, which is, in practice, more important and more difficult to address than the bias between a surrogate method and a reference method.

To our knowledge, this is the first time a single procedure brings together and applies the results of Hedberg and Ayers^23^, Shukla^24^, Shoukri^31^, and Linnet^27^, and provides a theoretical basis for statistics related to accuracy, precision, and Deming regression, respectively. This study also implemented analytical methods, bootstrapping, and easily interpretable graphical outputs. Most importantly, although each function in our package can be used independently (examples are detailed in the package documentation), we have created a coordinating function that allows researchers to use a single command to generate a complete report in plain text, HTML, or PDF from nothing but an Excel or similar file containing data in a data frame. The elements in Figure 1, for instance, were extracted from the report generated using the following command:


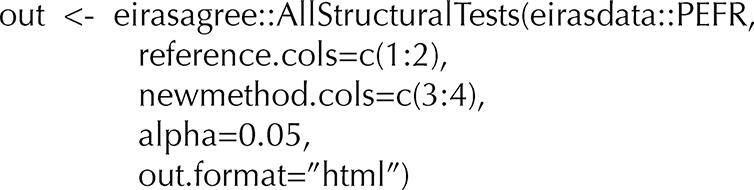


In addition, eirasagree provides treatment for repeated measures, which is not done by the other mentioned packages. It is common for researchers to take multiple measurements using the same technique when comparing a new method to replace an established one. Depending on whether unique or repeated measures are provided, eirasagree calculates the value of lambda and automatically uses it in subsequent tests. This feature enhances the package’s ability to handle repeated measurements effectively.

One of the most significant criticisms of both the traditional Bland-Altman plot method and the discussed packages is the reliance on visual inspection for decision-making. In this regard, eirasagree innovates by automating the recognition of lines or points within the regions of bands or ellipses, providing decision indicators for the users. This eliminates the subjective aspect of visual interpretation and increases objectivity in the decision-making process.

Another innovative concept of line translations was also introduced, allowing the assessment of precision and bisector agreement even in the presence of unequal means between two measurement techniques. Biased techniques that provide equal precision and agreement may still be useful with a simple calibration or correction. Reversely, if a surrogate technique is unbiased but less precise, it could be eligible as a screening step; however, if this imprecision imposes risks to patients, the technique must be reviewed. In essence, the decomposition of accuracy, precision, and agreement with the bisector line analysis can guide researchers in determining where to focus their efforts to improve a new technique when full equivalence is not achieved.

Notably, the axes used in the precision test are the same as those used in the original Bland-Altman plot method. Contrary to the belief of many users, the original method, even under optimal conditions, aims to compare the precision of two measurement techniques, not their equivalence. However, this comparison is not possible because the original Bland-Altman bands (i.e., the so-called limits of agreement, LoA) do not represent a confidence interval; they merely correspond to the limits of a Chebyshev interval^18,19^. Chebyshev’s inequality theorem provides information about the percentage of data that is guaranteed to fall within a given interval, regardless of probability distribution. For instance, in a normal distribution, approximately 95% of the data falls within plus or minus two standard deviations around the mean, while according to Chebyshev’s theorem, a minimum of 75% is guaranteed for any distribution. For comparison, Figure 1 illustrates the original Bland-Altman bands and highlights that decision-making cannot be solely based on whether or not the majority of points fall within the bands, because this is always the case. Additionally, these bands cannot provide information about any regression slopes, as they are always horizontal. The correct approach is to use the hyperbolic-shaped 95% confidence band, as shown in the precision tests on Figures 1, 2, 3, 4, and 5, which allows for the assessment of the existence of a slope-zero line, considering the precision between the two measurement methods. These bands can be inclined depending on the precision relationship of the methods, thus leading to the rejection of the null hypothesis of precision equivalence.

To compare two techniques, non-rejection of the null hypothesis is not enough, and the acceptance of equivalence (the acceptance of the null hypothesis) is conceptually necessary. Power computation obtained from a sample *a posteriori* is meaningless^46^, which means that planning sample size along with study design *a priori* is crucial to preserve statistical power. Budd et al.^47^ proposed at least 100 observations to claim the consistency of a candidate measurement procedure applicable to different populations (item 6.3, page 12). This number lowered to 40 after an analysis in more controlled laboratory conditions (item 7.2, page 15). However, this same source deals with more than a measure of each technique from the same patient, with average or median (which we disagree): it affects the computation of λ, wastes information, and, consequently, raises an ethical problem when invasive techniques are under assessment. Linnet also approached this issue, stating that sample sizes between 40 and 100 usually need to be reconsidered^48^ and that the ideal number depends on the quotient between the maximum and minimum measurements, proposing numbers ranging from small sample sizes to those in the order of 500 pairs of measurements (with mention to numbers up to thousands). Some classic Bland and Altman examples applied here and in many other published studies may be below the limit and only allow the rejection/non-rejection of null hypotheses, lacking power to define true equivalence along the three statistical steps presented in this study.

## CONCLUSION

By preserving Bland and Altman’s principle of graphical communication and implementing robust and suitable inferential statistics, it is possible to test whether two techniques have full equivalence. This approach decomposes equivalence into accuracy, precision, and agreement for measurement techniques, which helps find the source of problems when full equivalence does not verify, making it possible to fix new techniques. The use of the selected statistical methods using R provides automatized and standardized outputs of an otherwise complex calculation, allowing for better communication among researchers.
